# Whole-lesion ADC histogram and texture analysis in predicting recurrence of cervical cancer treated with CCRT

**DOI:** 10.18632/oncotarget.21374

**Published:** 2017-09-28

**Authors:** Jie Meng, Lijing Zhu, Li Zhu, Li Xie, Huanhuan Wang, Song Liu, Jing Yan, Baorui Liu, Yue Guan, Jian He, Yun Ge, Zhengyang Zhou, Xiaofeng Yang

**Affiliations:** ^1^ Department of Radiology, Nanjing Drum Tower Hospital, The Affiliated Hospital of Nanjing University Medical School, Nanjing, 210008, China; ^2^ The Comprehensive Cancer Centre of Drum Tower Hospital, The Affiliated Hospital of Nanjing University Medical School, Nanjing, 210008, China; ^3^ School of Electronic Science and Engineering, Nanjing University, Nanjing, 210046, China; ^4^ Department of Radiation Oncology and Winship Cancer Institute, Emory University, Atlanta, Georgia 30322, USA

**Keywords:** diffusion weighted imaging, magnetic resonance imaging, uterine cervical neoplasms, histogram analysis, texture analysis

## Abstract

**Purpose:**

To explore the value of whole-lesion apparent diffusion coefficient (ADC) histogram and texture analysis in predicting tumor recurrence of advanced cervical cancer treated with concurrent chemo-radiotherapy (CCRT).

**Methods:**

36 women with pathologically confirmed advanced cervical squamous carcinomas were enrolled in this prospective study. 3.0 T pelvic MR examinations including diffusion weighted imaging (b = 0, 800 s/mm^2^) were performed before CCRT (pre-CCRT) and at the end of 2nd week of CCRT (mid-CCRT). ADC histogram and texture features were derived from the whole volume of cervical cancers.

**Results:**

With a mean follow-up of 25 months (range, 11 ∼ 43), 10/36 (27.8%) patients ended with recurrence. Pre-CCRT 75th, 90th, correlation, autocorrelation and mid-CCRT ADC_mean_, 10th, 25th, 50th, 75th, 90th, autocorrelation can effectively differentiate the recurrence from nonrecurrence group with area under the curve ranging from 0.742 to 0.850 (P values range, 0.001 ∼ 0.038).

**Conclusions:**

Pre- and mid-treatment whole-lesion ADC histogram and texture analysis hold great potential in predicting tumor recurrence of advanced cervical cancer treated with CCRT.

## INTRODUCTION

Cervical cancer is the fourth most common gynecologic malignancy, and the fourth leading cause of cancer related deaths in women all over the world [1]. Although the standard treatment concurrent chemo-radiotherapy (CCRT) greatly extended the overall survival time of women with advanced diseases, approximately 40% of those patients would undergo recurrence [2, 3]. If there is a reliable prognostic biomarker before or at early stage of therapy, individualized therapeutic regimens can be developed, and more intensive follow-up or clinical trials can be under consideration for patients with probably poor prognosis.

The predictive efficiency of morphological indexes such as maximal tumor diameter (MTD) seemed limited based on previous studies, since treatment induced morphologic alterations occurred after tumor molecular and biological changes [4]. Functional sequences such as diffusion weighted (DW) imaging has been incorporated into routine protocols of pelvic MR imaging nowadays. It measures water molecular diffusion in terms of apparent diffusion coefficient (ADC) value and brings a better understanding into tumor microstructures noninvasively [5]. It was reported that cervical cancers with lower pre-treatment ADC values were at a higher risk of recurrence [6, 7]. And a lower rise of ADC values in early stage of CCRT may help predict a poor prognosis in patients with cervical cancers [8, 9].

However, only mean ADC value obtained from one region of interest (ROI) was used in most previous studies. A whole-lesion histogram-based approach could derive a series of features and reflect the microstructural heterogeneity of tumors by classifying portions with different diffusivities [10]. Some retrospective studies have shown that pretreatment ADC_mean_ and ADC percentiles were significantly lower in cervical cancers with recurrence compared to those without recurrence [4, 11–12].

Texture analysis based on ADC maps is an emerging modality to extract texture features describing local and regional relationships between pixels within the ROIs, which can better reflect intratumoral heterogeneity [13]. A recent study demonstrated the efficacy of texture analysis for predicting treatment response to chemoradiotherapy in nasopharyngeal carcinoma [14]. To the best of our knowledge, applications of this technique in predicting the prognosis of cervical cancer have not been reported yet.

Hence, the purpose of this study was to investigate whether pre- and mid-treatment ADC parameters including histogram features and texture features can serve as effective biomarkers for predicting tumor recurrence of patients with cervical cancers treated with CCRT.

## RESULTS

### Follow-up results

During a mean follow-up of 25 months (range, 11 ∼ 43), 10 patients (10/36, 27.8%; mean age, 51.1 years) were classified into the recurrence group (5 local recurrences, 5 deaths caused by cervical cancer), and the remaining 26 patients (26/36, 72.2%; mean age, 53.5 years) belonged to the nonrecurrence group. FIGO stages in the recurrence group included IIA (n = 1), IIB (n = 3), IIIB (n = 3) and IVA (n = 3). FIGO stages in the nonrecurrence group included IIA (n = 5), IIB (n = 10), IIIA (n = 2), IIIB (n = 5) and IVA (n = 4). The recurrence incidence of high FIGO staging (stage III or above) group (6/17, 35.3%) seemed higher than that of low FIGO staging (stage IIA, IIB) group (4/19, 21.0%), yet without significance (P = 0.281). FIGO staging is not sufficient to accurately predict therapeutic response or prognosis of cervical cancer [15]. Hence, we did not use FIGO staging as a prognostic factor in this study.

Since none of the patients underwent surgical lymphadenectomy, we used size (short axis > 8–10 mm) as the main imaging criterion for discriminating normal from metastatic pelvic lymph node (PLN). Among 11 patients in PLN positive group, 6 patients had tumor recurrence. Among 25 patients in PLN negative group, 4 patients had tumor recurrence. The incidence of recurrence was significantly higher in the PLN positive group compared to the PLN negative group (54.5% vs. 16.0%, P = 0.026). Due to the low sensitivity of determining lymph node status on MR imaging, we also did not use lymph node status as a prognostic factor in this study.

**Figure 1:**
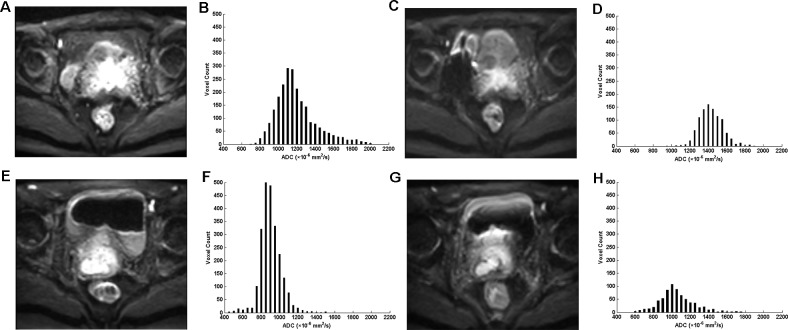
Diffusion weighted (DW) magnetic resonance images (b = 800 s/mm^2^) and the corresponding apparent diffusion coefficient (ADC) histograms of two representative patients **(A-D)** A 52-year-old woman with advanced cervical cancer (the international federation of gynecology and obstetrics (FIGO) stage IIIB) receiving concurrent chemo-radiotherapy (CCRT) achieved complete remission in the follow-up process. **(E-H)** A 53-year-old woman with advanced cervical cancer (FIGO stage IIIB) receiving CCRT died six months after CCRT completion. (A, E) DW images before CCRT; (B, F) ADC histograms before CCRT show significant difference between the two patients; (C, G) DW images at the end of 2nd week of CCRT; (D, H) ADC histograms at the end of 2nd week of CCRT show significant difference between the two patients.

Representative cases of cervical cancer that showed significant difference of ADC histogram between patients with different prognosis before and at the end of 2nd week of CCRT are shown in Figure 1.

### Predictive value of morphological parameters

As shown in Table 1, pre- and mid-CCRT MTD, area and volume showed no statistical differences between the recurrence and nonrecurrence group (all P > 0.05).

**Table 1:** Comparison of pre- and mid-CCRT morphological parameters between the recurrence and nonrecurrence group of patients with cervical cancers treated with CCRT

Time point	Parameters	Nonrecurrence group	Recurrence group	P value
	MTD	50.88 ± 12.18	46.43 ± 12.69	0.338
pre-CCRT	area	7158.69 ± 4799.17	4629.76 ± 3682.28	0.143
	volume	37722.58 ± 24177.35	25585.26 ± 22700.66	0.179
	MTD	38.19 ± 14.53	39.01 ± 15.32	0.891
mid-CCRT	area	3784.85 ± 3888.81	2799.83 ± 3168.86	0.511
	volume	19566.62 ± 20111.85	14221.14 ± 15742.34	0.488

Note: Data are presented as mean ± standard deviation. CCRT = concurrent chemo-radiotherapy; MTD = maximal tumor diameter.

### Predictive value of ADC first-order statistics

As shown in Table 2, pre- and mid-CCRT ADC_mean_ of the recurrence group were significantly lower than those of the nonrecurrence group (P = 0.018, 0.002, respectively). ROC analysis showed that pre-CCRT ADC_mean_ could not predict prognosis (P = 0.061) while mid-CCRT ADC_mean_ had a good prognostic value (P = 0.006).

**Table 2:** Comparison of pre- and mid-CCRT ADC histogram features between the recurrence and nonrecurrence group of patients with cervical cancers treated with CCRT and their diagnostic performance

Time point	Parameters	Nonrecurrence group	Recurrence group	P value^#^	Sensitivity	Specificity	Accuracy	AUC (95% CI)	P value^+^
pre-CCRT	ADC_mean_	1064.76 ± 67.44	983.71 ± 127.25	0.018^*^	84.6	60.0	77.8	0.704 (0.495, 0.913)	0.061
	5th	726.38 ± 134.77	699.90 ± 170.40	0.627	65.4	50.0	61.1	0.521 (0.292, 0.751)	0.846
	10th	784.54 ± 121.82	752.60 ± 160.58	0.524	69.2	50.0	63.9	0.538 (0.313, 0.764)	0.724
	25th	876.00 ± 109.89	838.10 ± 145.96	0.404	53.8	70.0	58.3	0.606 (0.395, 0.817)	0.331
	50th	999.31 ± 90.45	943.10 ± 136.08	0.157	57.7	70.0	61.1	0.633 (0.419, 0.846)	0.223
	75th	1201.46 ± 73.89	1090.10 ± 125.99	0.002^**^	96.2	50.0	83.4	0.781 (0.602, 0.960)	0.010^*^
	90th	1448.50 ± 146.79	1273.30 ± 122.28	0.002^**^	69.2	90.0	75.0	0.819 (0.680, 0.959)	0.003^*^
	skewness	2.23 ± 1.11	1.91 ± 0.79	0.411	69.2	60.0	66.6	0.615 (0.405, 0.825)	0.289
	kurtosis	12.93 ± 9.22	11.14 ± 5.05	0.567	38.5	80.0	50.0	0.535 (0.327, 0.742)	0.751
	entropy	6.23 ± 0.58	6.10 ± 0.27	0.507	69.2	70.0	69.4	0.658 (0.480, 0.835)	0.148
mid-CCRT	ADC_mean_	1333.24 ± 123.96	1171.21 ± 108.11	0.002^**^	90.0	66.7	82.8	0.822 (0.662, 0.983)	0.006^*^
	5th	975.60 ± 204.60	833.33 ± 169.51	0.080	60.0	88.9	69.0	0.700 (0.503, 0.897)	0.090
	10th	1054.70 ± 184.67	902.44 ± 146.32	0.038^*^	55.0	88.9	65.5	0.711 (0.518, 0.905)	0.073
	25th	1161.75 ± 159.14	1006.89 ± 132.06	0.017^*^	55.0	88.9	65.5	0.764 (0.581, 0.947)	0.025^*^
	50th	1292.55 ± 135.23	1147.67 ± 116.64	0.010^*^	90.0	66.7	82.8	0.794 (0.615, 0.974)	0.012^*^
	75th	1472.70 ± 121.57	1297.00 ± 111.84	0.001^**^	65.0	100.0	75.9	0.850 (0.712, 0.988)	0.003^*^
	90th	1665.95 ± 140.67	1482.67 ± 196.97	0.008^*^	85.0	77.8	82.8	0.822 (0.621, 1.000)	0.006^*^
	skewness	1.38 ± 1.09	1.14 ± 1.30	0.613	85.0	44.4	72.4	0.567 (0.320, 0.814)	0.572
	kurtosis	8.99 ± 6.82	8.17 ± 7.04	0.769	50.0	77.8	58.6	0.561 (0.324, 0.798)	0.604
	entropy	5.75 ± 0.93	5.68 ± 0.69	0.844	30.0	100.0	51.7	0.572 (0.364, 0.780)	0.540

Note: Data are presented as mean ± standard deviation; ADC_mean_ and all percentile values are in unit of × 10^−6^ mm^2^/s; CCRT, concurrent chemo-radiotherapy; ADC, apparent diffusion coefficient; AUC, area under receiver operating characteristic (ROC) curve; CI, confidence interval; P value^#^, with independent samples t test; P value^+^, with ROC analysis; ^*^ P value < 0.05; ^**^ P value < 0.005 (adjusted significance level).

Before CCRT, only two ADC percentiles (75th and 90th) differed significantly between patients with different prognosis (P = 0.002). After two weeks of treatment, up to five ADC percentiles (10th, 25th, 50th, 75th, 90th) were significantly lower in the recurrence group (all P < 0.05).

Nevertheless, ADC histogram shape related parameters, including skewness, kurtosis and entropy, showed no statistical differences between patients with different prognosis (all P > 0.05).

### Predictive value of ADC texture features

As shown in Table 3, before CCRT, correlation and autocorrelation showed significant differences between patients with different prognosis (P = 0.026, 0.006, respectively). After 2 weeks of CCRT, only one texture feature autocorrelation could distinguish between the recurrence and nonrecurrence group (P = 0.002).

**Table 3:** Comparison of pre- and mid-CCRT ADC texture features between the recurrence and nonrecurrence group of patients with cervical cancers treated with CCRT and their diagnostic performance

Time point	Parameters	Nonrecurrence group	Recurrence group	P value^#^	Sensitivity	`Specificity	Accuracy	AUC (95% CI)	P value^+^
pre-CCRT	correlation	8.76 (6.17, 12.59)	23.98 (12.73, 34.98)	0.026^*^	80.0	73.1	75.0	0.742 (0.547, 0.938)	0.026^*^
	autocorrelation	88689.45 (73619.12, 101094.67)	62330.77 (55900.31, 71437.64)	0.006^**^	76.9	80.0	77.8	0.792 (0.598, 0.986)	0.007^*^
	entropy(H)	11.31 (9.07, 12.18)	11.30 (10.43, 11.75)	0.903	100.0	34.6	52.8	0.515 (0.329, 0.702)	0.888
	homogeneity	8561.20 (6976.18, 10457.48)	9823.74 (6774.34, 10661.50)	0.689	50.0	73.1	66.9	0.546 (0.317, 0.776)	0.672
mid-CCRT	correlation	14.87 (6.65, 24.34)	23.78 (20.25, 39.13)	0.116	77.8	60.0	65.5	0.689 (0.472, 0.906)	0.109
	autocorrelation	119709.75 (104566.62, 137939.81)	93120.65 (73795.97, 106326.00)	0.002^**^	95.0	66.7	86.2	0.844 (0.686, 1.000)	0.003^*^
	entropy(H)	10.20 (7.55, 11.50)	9.92 (8.69, 11.66)	0.982	50.0	66.7	55.2	0.506 (0.289, 0.722)	0.962
	homogeneity	7150.19 (6082.49, 8873.28)	9071.10 (7078.09, 10696.41)	0.167	66.7	75.0	72.4	0.667 (0.444, 0.889)	0.157

Note: Data are presented as median (interquartile range); Correlation and homogeneity values are in unit of × 10^−5^ mm^2^/s and autocorrelation are in unit of × 10^−6^ mm^2^/s; CCRT, concurrent chemo-radiotherapy; ADC, apparent diffusion coefficient; AUC, area under receiver operating characteristic (ROC) curve; CI, confidence interval; P value^#^, with independent samples t test; P value^+^, with ROC analysis; ^*^ P value < 0.05; ^**^ P value < 0.013 (adjusted significance level).

After performing test correction, pre-CCRT 75th, pre-CCRT 90th, pre-CCRT autocorrelation, mid-CCRT ADC_mean_, mid-CCRT 75th and mid-CCRT autocorrelation could still distinguish patients with different prognosis, and we included those parameters in the multivariate stepwise logistic regression analysis. The results showed that the two independent differentiating variables were pre-CCRT 90th (P = 0.0002) and mid-CCRT 75th (P = 0.0005). Using the cutoff of 1376.50 × 10^−6^ mm^2^/s for the pre-CCRT 90th percentile, ADC histogram analysis had an accuracy of 86.1 % and made accurate predictions for 31 of the 36 patients. Using the cutoff of 1428.50 × 10^−6^ mm^2^/s for the mid-CCRT 75th percentile, ADC histogram analysis had an accuracy of 83.3 % and made accurate predictions for 30 of the 36 patients.

**Figure 2:**
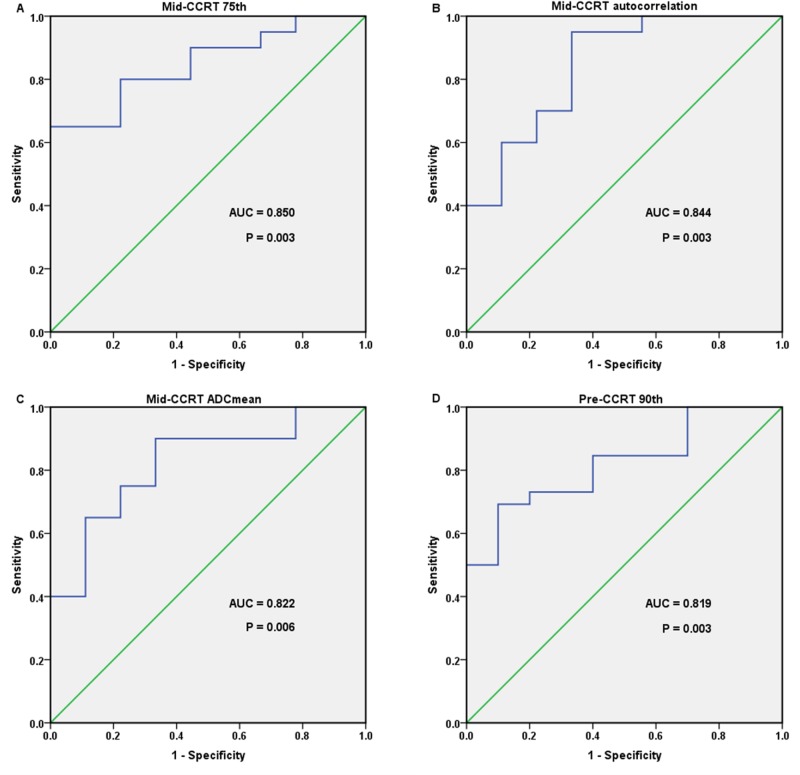
Receiver operating characteristic (ROC) curves of four important parameters that showed the largest area under the curves (AUCs) in the prediction of cervical cancer recurrence treated with concurrent chemo-radiotherapy (CCRT) **(A)** mid-CCRT 75th, with an AUC of 0.850; **(B)** mid-CCRT autocorrelation, with an AUC of 0.844; **(C)** mid-CCRT ADC_mean_, with an AUC of 0.822; **(D)** pre-CCRT 90th, with an AUC of 0.819.

**Figure 3:**
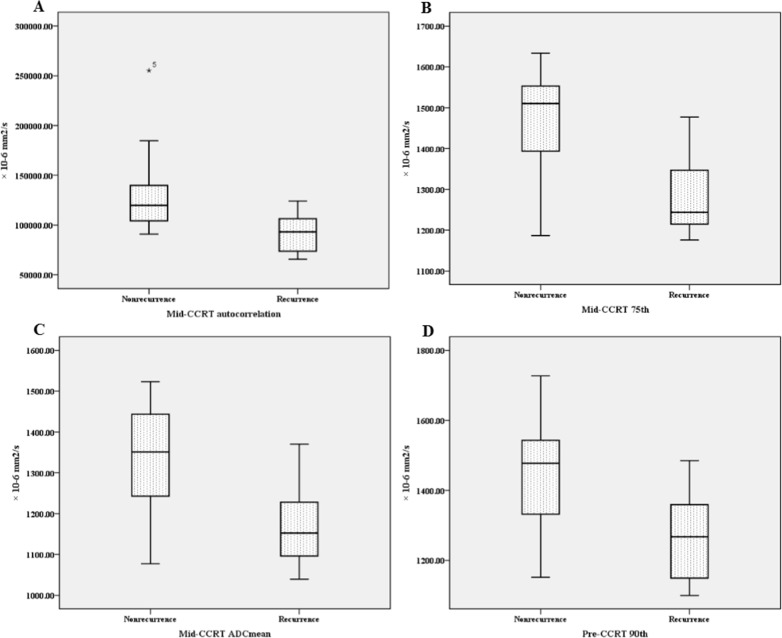
Box-and-whisker plots of four important parameters which all differed significantly between cervical cancer patients with different prognosis after undergoing concurrent chemo-radiotherapy (CCRT) **(A)** mid-CCRT autocorrelation; **(B)** mid-CCRT 75th; **(C)** mid-CCRT ADC_mean_; **(D)** pre-CCRT 90th.

Mid-CCRT 75th, autocorrelation, ADC_mean_ and pre-CCRT 90th showed the largest AUCs in the prediction of tumor recurrence; the AUCs were 0.850, 0.844, 0.822 and 0.819, respectively (all P < 0.01). The ROC curves as well as Box-and-whisker plots of those important parameters were shown as Figure 2 and Figure 3.

### Predictive value of parameters change rates

The change rates of three morphological parameters showed no significant differences between the recurrence and nonrecurrence group. Similarly, no change rate index of ADC histogram or texture features at the end of 2nd week of CCRT was proved useful in prognostic prediction (all P > 0.05). The details were shown in the Supplementary Table 1.

### Inter-observer reproducibility

As shown in Table 4, the inter-observer agreements achieve at least good for all the ADC histogram and texture parameters.

**Table 4:** Inter-observer agreements for the ADC histogram and texture parameters of cervical cancer patients

Parameter	ICC (pre-CCRT)	ICC (mid-CCRT)
ADC_mean_	0.86 (0.79 - 0.96)	0.94 (0.77 - 0.98)
5th	0.99 (0.97 - 0.99)	0.99 (0.96 - 0.99)
10th	0.99 (0.98 - 0.99)	0.99 (0.96 - 0.99)
25th	0.98 (0.94 - 0.99)	0.89 (0.74 - 0.98)
50th	0.94 (0.78 - 0.98)	0.96 (0.85 - 0.99)
75th	0.79 (0.62 - 0.94)	0.89 (0.77 - 0.97)
90th	0.79 (0.611 - 0.895)	0.86 (0.77 - 0.97)
skewness	0.85 (0.41 - 0.96)	0.70 (0.22 - 0.93)
kurtosis	0.94 (0.72 - 0.98)	0.75 (0.03 - 0.94)
entropy	0.61 (0.36 - 0.90)	0.92 (0.69 - 0.98)
correlation	0.93 (0.73 - 0.98)	0.88 (0.66 - 0.97)
autocorrelation	0.86 (0.70 - 0.97)	0.97 (0.88 - 0.99)
entropy(H)	0.88 (0.64 - 0.97)	0.96 (0.83 - 0.99)
homogeneity	0.71 (0.63 - 0.93)	0.93 (0.74 - 0. 98)

Note: ADC, apparent diffusion coefficient; CCRT, concurrent chemo-radiotherapy; ICC, intra-class correlation coefficient; ICC, 0.00 - 0.40 poor; 0.41 - 0.60 fair; 0.61 - 0.80 good; 0.81 - 1.00 excellent. Contents in parentheses are 95% confidence interval.

## DISCUSSION

In this study, we demonstrated that ADC histogram and texture parameters before and at the early stage of CCRT could predict tumor recurrence of patients with advanced cervical cancers.

Morphological parameters MTD, area, volume and their change rates between pre- and mid-CCRT showed no statistical differences between the recurrence and nonrecurrence group, indicating that the prognostic value of ADC histogram and texture features is superior to traditional morphological parameters for cervical cancer underwent CCRT.

ADC_mean_ is the most basic and commonly used parameter. We found that patients with tumor recurrence showed lower pre-CCRT ADC_mean_, but we found it helpless to predict long-term outcome. Erbay et al. [12] also found that ADC_mean_ before CCRT was significantly lower in cervical cancer patients with tumor recurrence compared with those without recurrence. However, Heo et al. [4] reported just the opposed results. Somoye et al. [8] and Bae et al. [9] also reported that pre-treatment ADC_mean_ did not show any differences between good and poor long-term prognoses in cervical cancers. This discrepancy may be due to the following explanations: (1) the ROI placement with or without necrotic area in the tumors, while our study drew VOIs of the whole tumor (including cystic and necrotic areas) instead of traditional ROI (only one slice); (2) tumor heterogeneity, while our study contains various ADC histogram and texture parameters which can reflect the tumor heterogeneity. For example, the low and high ADC percentiles represent different components of the tumor and higher autocorrelation indicates less heterogeneous the tumor is; and (3) different MR protocols and parameters, which need to be standardized in the future. Thus, pre-treatment ADC_mean_ may not be an eligible prognostic biomarker for cervical cancer. Fortunately, we found that mid-CCRT ADC_mean_ had a good prognostic value for patients with cervical cancers. Bae et al. [9] also reported that mid-treatment ADC_mean_ was significantly lower in tumor recurrence group and could serve as a useful prognostic biomarker.

Our results showed that pre-CCRT 75th and 90th ADC percentiles were significantly lower in the recurrence group. Erbay et al. [12] also found that patients with tumor recurrence showed lower pre-treatment 75th and 90th ADC percentiles, which were independent prognostic factors for both overall survival and disease-free survival. It was raised that lower 75th and 90th ADC percentiles might represent less cystic or necrotic regions within the tumor, namely a greater proportion of solid tumor may indicate a higher risk of recurrence. After 2 weeks of CCRT, more ADC percentiles showed significant differences between different prognosis in our study.

Another interesting finding about ADC percentiles was that the high ADC percentiles (75th, 90th) showed higher AUCs than the low ADC percentiles (10th, 25th, 50th). And pre-CCRT 90th and mid-CCRT 75th were the two independent differentiating variables. Heo et al. [4] also demonstrated pre-treatment 75th ADC percentile was a significant predictor for cervical cancer recurrence while 25th and 50th had no prognostic value. We speculated that the high ADC percentiles are related with tumor necrosis while the low percentiles are mainly determined by tumor cell density. Necrotic tumors are prone to progression and recurrence after chemo-radiotherapy on account of hypoxia and poor perfusion [16]. However, other tumors did not follow this rule. For instance, Song et al. [17] reported that 5th ADC percentile was the most promising parameter for differentiating true progression from pseudoprogression of glioblastomas treated with CCRT. And Donati et al. [18] found that 10th ADC percentile correlated with Gleason score better than other ADC parameters in prostate cancer.

ADC texture analysis is mainly applied in distinction between benign and malignant tumors and differentiating tumor grades [19–24], seldom in prognosis prediction. We found that pre-CCRT correlation and autocorrelation could differentiate between the recurrence and nonrecurrence group. Yun et al. [23] reported that pre-treatment correlation could help to determine intratumoral spatial heterogeneity of necrotic patterns, which may be the basis for its predictive value. Autocorrelation reflected the similarity of the ADC pairs. The higher autocorrelation was, the less heterogeneous the tumor was. In this study, both pre- and mid-CCRT autocorrelation were significantly lower in the recurrence group, indicating that tumor recurrence was associated with greater heterogeneity within the tumor.

Due to our limited sample size (only 36 patients), we used all the cases as modeling group. For internal validation, we retrospectively analyzed 10 patients with advanced cervical squamous cell carcinomas treated with CCRT. All those 10 patients underwent routine pelvic MR examination including DWI before CCRT. During follow-up (median, 20 months; range, 17 ∼ 34 months), 3 patients reported pelvic tumor recurrence and the others achieved complete remission. We performed ADC histogram and texture analysis for pre-CCRT MR imaging and the relevant parameters for prognosis are listed in the Supplementary Table 2. According to our thresholds established from the previous 36 modeling patients, we found that 9/10 (90%) patients were accurately predicted by using 75th percentile and 8/10 (80%) patients were accurately predicted by using 90th percentile or correlation. Since those 10 patients did not undergo MR examination 2 weeks after the initiation of CCRT, the predictive power of mid-CCRT parameters could not be validated. In the future, we will continue to collect more cases for further internal validation, and try our best to perform external validation.

Our study had several limitations. Firstly, only 36 patients with squamous carcinomas were included, which might cause some bias. Yet it was enough for a pilot prospective study. A larger cohort with multiple pathological subtypes is required to confirm the findings in this study. Secondly, the follow-up was relatively short and there might be a few additional recurrences with a longer follow-up. Thirdly, only two b values of 0 and 800 s/mm^2^ were applied in the DW sequence of the current study and the use of b0 may cause inaccuracy when calculating ADC values. Acquiring DW images using a range of b values or using a multi-exponential fit could provide more accurate estimation of tumor cellularity [24].

In summary, whole-lesion ADC histogram and texture analysis hold great potential in predicting tumor recurrence in cervical cancer treated with CCRT. Pre-CCRT 90th and mid-CCRT 75th were considered to be the two most important and independent factors associated with the prognosis. This technique could be applied to identify patients at higher risks of recurrence before or at early stage of treatment, which may help to optimize therapeutic regimens or undertake more intensive follow-ups.

## MATERIALS AND METHODS

### Patients

**Figure 4:**
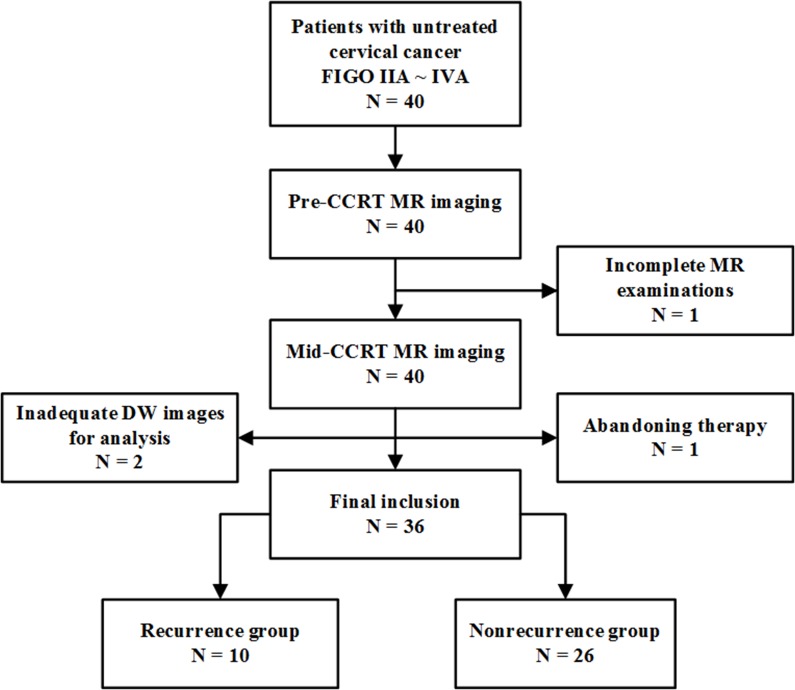
Flowchart of the study population FIGO = International Federation of Gynecology and Obstetrics, CCRT = concurrent chemo-radiotherapy.

This prospective study was approved by the local ethics committee, and written informed consent was obtained from all patients. From April 2013 to August 2015, 40 women with histologically proven untreated cervical cancer (International Federation of Gynecology and Obstetrics (FIGO) IIA ∼ IVA), scheduled to undergo CCRT were consecutively recruited. 4 patients were excluded for the following reasons: patients who failed to complete MR examinations or the full course of CCRT due to personal reasons (n = 2); poor quality of DW images due to patient motion or magnetic susceptibility artifacts (n = 2). Finally, a total of 36 women (all cervical squamous carcinomas, mean age, 53 years; age range, 25 ∼ 77) were included in this study (Figure 4). The FIGO stage was determined by clinical evaluation consensus with MRI evaluation. The FIGO stages included IIA (n = 6), IIB (n = 13), IIIA (n = 2), IIIB (n = 8), IVA (n = 7).

All patients underwent 5-weeks external beam radiation therapy (EBRT) followed by 3-weeks intracavitary brachytherapy (ICBT). EBRT was delivered to the whole pelvis at 1.8-2.0 Gy daily with a total dose of 45-50 Gy. ICBT was given to point A at a fraction dose of 5 Gy with a total dose of 30-40 Gy. Six cycles of weekly nedaplatin or four cycles of bi-weekly nedaplatin plus paclitaxel/docetaxel was given concomitantly.

### MR imaging

All patients were scheduled to undergo MR examination twice: within 2 weeks before CCRT (pre-CCRT) and at the end of 2nd week during CCRT (mid-CCRT). All examinations were performed on a 3.0-T MR scanner (Ingenia 3.0 T, Philips Healthcare, Best, the Netherlands) with a 16-channel torso phased-array body coil. The scan range was set to cover the whole pelvis. All patients were asked to take clyster 2-3 hours before the MR examination in order to reduce artifacts induced by gas and feces within the rectum. The standard MR scan protocol was kept identical each time as follows: axial T1-weighted turbo spin-echo (TSE) sequence (repetition time (TR) = 500 ms, echo time (TE) = 12 ms, matrix size = 282 × 400, field of view (FOV) = 35 cm × 40 cm, slice thickness = 4 mm, intersection gap = 0.5 mm, number of signal averages (NSA) = 1), axial T2-weighted TSE sequence (TR = 4,500 ms, TE = 90 ms, matrix size = 308 × 402, FOV = 20 cm × 24 cm, slice thickness = 4 mm, intersection gap = 0.5 mm, NSA = 1), sagittal T2-weighted TSE sequence (TR = 4500 ms, TE = 90 ms, matrix size = 480 × 354, FOV = 20 × 24 cm, slice thickness = 4 mm, intersection gap = 0.5 mm, NSA = 1), axial DW free-breathing spin-echo echo-planar-imaging sequence (TR = 3523-6000 ms, TE = shortest ms, matrix size = 132 × 157, FOV = 24 cm × 24 cm, slice thickness = 4 mm, intersection gap = 1 mm, NSA = 2, b value = 0 and 800 s /mm^2^). axial and sagittal contrast enhancerd-T1 high resolution isotropic volume examination (e-THRIVE) sequences (TR = shortest ms, TE = shortest ms, matrix size = 256 × 194, FOV = 30 × 40cm, slice thickness = 1.5 mm, intersection gap = 0 mm, NSA = 1) was also acquired after intravenous injection of 0.2 mL per kilogram of body weight Gadodiamide (Omniscan, GE Healthcare, Shanghai, China).

### Image analysis

Axial DW images were loaded into a workstation (Extended MR Workspace 2.6.3.4; Philips Medical Systems, Best, the Netherlands) and ADC maps were generated automatically using the mono-exponential model. Then DW images and the corresponding ADC maps were imported into our in-house software (Image Analyzer 1.0, Nanjing, China). The whole-lesion analysis was performed by two radiologists (^*^BLINDED^*^, with 5 and 8 years’ experience in gynecological imaging, respectively) independently, who were unaware of patients’ outcomes. Cervical cancers presented as hypointense on ADC maps, hyperintense on T2-weighted and DW images, with remarkable enhancement on e-THRIVE images. ROIs were manually drawn slice by slice on the DW images (b = 800 s/mm^2^) to include as much tumor area as possible including cystic and necrotic areas with reference to other sequences. The outlines of ROIs drawn on each slice would be automatically copied to the exact same location of the corresponding ADC maps in our software in real time.

All the ROIs that covered the entire tumor were selected to derive the volume of interest (VOI). Then a button was clicked in our software, and 3 kinds of parameters were generated automatically: (1) morphological parameters, including maximal tumor diameter (MTD), area (the area of all the ROIs) and volume; (2) ADC histogram features, including ADC_mean_, ADC percentiles (5th, 10th, 25th, 50th, 75th and 90th percentiles), skewness, kurtosis and entropy; (3) ADC texture features, including correlation, autocorrelation, entropy(H) and homogeneity.

ADC histogram features have been commonly described in previous studies [10, 25]. Such parameters refer to the frequency of the intensity of gray levels without considering the spatial relationship between them. Texture features in our study were derived from the gray level co-occurrence matrix (GLCM) within the VOI. A GLCM is a constructed matrix in which P(i, j) describes the probability of a pair of gray levels (i and j) occurring in an image. Every pair of gray levels is separated by a certain distance in a certain direction. In our work, the GLCMs were calculated with distance of one voxel and direction angles of 0, 45, 90 and 135, respectively. We took average values of GLCMs in the four directions as the final values of texture features. The formulas of texture features in this study were listed as follows:

$$\text{correlation}=\sum_{i=1}^{G}\sum_{j=1}^{G}\frac{\left(i-\mu )(j-\mu )P(i,j\right)}{\sigma^{2}}$$

$$\text{autocorrelation}=\sum_{i=1}^{G}\sum_{j=1}^{G}\text{ij}P(i,j)$$

$$\text{entropy}(H)=-\sum_{i=1}^{G}\sum_{j=1}^{G}P(i,j)\log_{2}\left[P(i,j)\right]$$

$$\text{homogeneity}=\sum_{i=1}^{G}\sum_{j=1}^{G}\frac{P(i,j)}{1+{\left(i-j\right)}^{2}}$$

Where G is the number of gray levels within the VOI, σ is the standard deviation of GLCM element, and μ is the mean of **P**(i, j). Definitions and demonstrations of ADC parameters above are shown in Table 5.

**Table 5:** Definitions and demonstrations of apparent diffusion coefficient (ADC) parameters

		Definition	Demonstration for a higher value of a parameter
ADC histogram features	skewness	a measure of the asymmetry of distribution	increased asymmetry from the normal distribution
	Kurtosis	a measure of the tailedness of distribution	a sharper peak and wider tails of the distribution of ADC values
	Entropy	a measure of the randomness of ADC values in an ADC map	more random distribution of gray levels, more heterogeneous
ADC texture features	entropy(H)	a measure of the randomness of ADC pairs in an ADC map considering the spatial information	more random distribution of paired gray levels, more heterogeneous
	homogeneity	a measure of the uniform of the ADC pairs	increased uniformity of the texture, less heterogeneous
	correlation	a measure of the linear dependencies of gray levels	increased uniformity of the texture, less heterogeneous
	autocorrelation	a measure of the similarity of the ADC pairs	a higher extent of similarity of ADC values, less heterogeneous

We also calculated change rates of all kinds of parameters by the following formulas:

MTD [area or volume] response (%) = (pre-CCRT MTD [area or volume] – mid-CCRT MTD [area or volume])/pre-CCRT MTD [area or volume] ^*^ 100.

ADC response (%) = |mid-CCRT ADC – pre-CCRT ADC|/pre-CCRT ADC ^*^ 100.

### Long-term outcome evaluation

After the completion of CCRT, follow-ups were performed 1 month, 3 months and afterwards every 6 months. Each visit contained medical records review, gynecological examinations and serum tumor markers detection. MR examinations were also performed for suspected tumor recurrence. Patients’ outcomes were divided into 2 groups: recurrence group, including recurrences, which were considered to be biopsy-proven or clinically diagnosed (by gynaecologists) replaces including pelvic recurrence and distant metastasis, or any lesion that was significantly enlarged at the latest follow-up, or deaths caused by cervical cancer; nonrecurrence group, that is, any patient who showed negative physical examination, negative tissue biopsy or negative imaging findings within the normal range of tumor markersin the follow-up process.

### Statistical analyses

Statistical analyses were performed using SPSS 22.0 software (SPSS Inc., Chicago, IL, USA). Independent samples t test was used to compare ADC histogram parameters and their change rates between the recurrence and nonrecurrence group. As some ADC texture parameters groups did not verify the normality assumption after performing Shapiro-Wilk test, Mann–Whitney U test was used to compare ADC texture parameters between the recurrence and nonrecurrence group. The predictive values of those parameters were tested by receiver operating characteristic (ROC) analysis, and were compared by multivariate stepwise logistic regression analysis. Two-tailed P values less than 0.05 were considered statistically significant. Dunn-Sidák correction was performed to adjust the significance level for ADC histogram parameters and texture features. Inter-observer reproducibility of ADC parameters was measured by calculating the intra-class correlation coefficient (ICC) with 95% confidence interval.

## SUPPLEMENTARY MATERIALS TABLES



## Abbreviations

CCRT: concurrent chemo-radiotherapy
MTD: maximal tumor diameter
DW: diffusion weighted
ADC: apparent diffusion coefficient
ROI: region of interest
FIGO: International Federation of Gynecology and Obstetrics
EBRT: external beam radiotherapy
ICBT: intracavitary brachytherapy
TR/TE: repetition time/echo time
FOV: field of view
VOI: volume of interest
GLCM: the gray level co-occurrence matrix
ROC: receiver operating characteristic
PLN: pelvic lymph node
AUC: area under the curve.
