# Supplementary material for: Whole-lesion ADC histogram and texture analysis in predicting recurrence of cervical cancer treated with CCRT
Source: Oncotarget. 2017 Sep 28;8(54):92442–53. doi: 10.18632/oncotarget.21374 (PMC5696195; doi:10.18632/oncotarget.21374)
Supplement: Supplementary file 1 [file oncotarget-08-92442-s001.pdf]

Whole-lesion ADC histogram and texture analysis in predicting recurrence of cervical cancer treated with CCRT

SUPPLEMENTARY MATERIALS

Supplementary Table 1: Comparison of pre- and mid-CCRT parameters' change rates between the recurrence and nonrecurrence group of patients with cervical cancers treated with CCRT and their diagnostic performance

Parameters	Nonrecurrence group	Recurrence group	P value[#]	Sensitivity	Specificity	Accuracy	AUC	P value[+]
MTD	25.45 ± 26.95	11.45 ± 39.73	0.356	44.4	50.0	48.3	0.589	0.451
area	23.88 ± 151.09	27.59 ± 73.64	0.945	88.9	35.0	51.7	0.572	0.540
volume	23.88 ± 151.09	28.45 ± 73.98	0.932	88.9	35.0	51.7	0.561	0.604
ADC_{mean}	25.57 ± 12.30	22.06 ± 20.15	0.638	75.0	66.7	69.3	0.611	0.346
5th	38.78 ± 33.19	21.96 ± 31.42	0.210	75.0	66.7	69.3	0.656	0.187
10th	37.04 ± 27.71	23.49 ± 28.18	0.236	75.0	66.7	69.3	0.628	0.278
25th	33.65 ± 21.93	23.51 ± 22.93	0.266	85.0	55.6	64.7	0.628	0.278
50th	29.50 ± 14.51	24.46 ± 19.07	0.439	75.0	66.7	69.3	0.633	0.258
75th	23.04 ± 10.68	22.01 ± 17.99	0.849	80.0	66.7	70.8	0.606	0.370
90th	16.12 ± 10.24	19.98 ± 23.57	0.538	66.7	60.0	62.1	0.539	0.741
skewness	40.17 ± 50.81	41.09 ± 72.30	0.969	90.0	44.4	58.6	0.572	0.540
kurtosis	31.82 ± 32.84	30.89 ± 53.09	0.954	65.0	77.8	73.8	0.594	0.423
entropy	7.75 ± 15.24	6.86 ± 11.63	0.878	45.0	77.8	67.6	0.511	0.925
correlation	129.49 ± 258.77	26.99 ± 122.29	0.314	65.0	66.7	66.2	0.628	0.278
autocorrelation	51.00 ± 72.06	62.13 ± 80.99	0.714	80.0	44.4	55.4	0.511	0.925
entropy(H)	9.84 ± 79.41	13.06 ± 13.76	0.402	55.0	66.7	63.1	0.500	1.000
homogeneity	5.40 ± 46.95	7.39 ± 19.67	0.904	88.9	35.0	51.7	0.578	0.509

Note: Data are presented as mean ± standard deviation; ADC, apparent diffusion coefficient; AUC, area under receiver operating characteristic

Supplementary Table 2: Aninternal validation set for the relevant parameters for prognosis before CCRT

Pre-CCRT parameters	75th	90th	correlation
**Cut-off value (recurrence)**	**< 1085.50**	**< 1376.50**	**> 12.36**
Patient 1 (recurrence)	996.00	1207.00	17.90
Patient 2 (recurrence)	861.00	1010.00	3.48*
Patient 3 (recurrence)	969.00	1138.00	17.50
Patient 4 (nonrecurrence)	1311.00	1459.00	2.47
Patient 5 (nonrecurrence)	1406.00	1568.00	7.26
Patient 6 (nonrecurrence)	1124.00	1380.00	8.99
Patient 7 (nonrecurrence)	1031.00*	1275.00*	7.24
Patient 8 (nonrecurrence)	1135.00	1296.00*	13.90*
Patient 9 (nonrecurrence)	1225.00	1528.00	4.52
Patient 10 (nonrecurrence)	1265.00	1456.00	2.25

Note: CCRT, concurrent chemo-radiotherapy; correlation values are in unit of $\times 10^{-5}$ mm^2/s and the others are in unit of $\times 10^{-6}$ mm^2/s; Patients 1 ~ 3: recurrence; Patients 4 ~ 10: nonrecurrence. 75th, 90th and correlation showed significant difference between the recurrence and nonrecurrence group after performing test correction. *, the prediction is incorrect.
